# Supplementary material for: PoreDesigner for tuning solute selectivity in a robust and highly permeable outer membrane pore
Source: Nat Commun. 2018 Sep 10;9:3661. doi: 10.1038/s41467-018-06097-1 (PMC6131167; doi:10.1038/s41467-018-06097-1)
Supplement: Supplementary file 1 — Supplementary Information [file 41467_2018_6097_MOESM1_ESM.pdf]

Chowdhury *et al.* **PoreDesigner for tuning solute selectivity in a robust and highly permeable outer membrane pore.**

Supplementary Methods

Isolation of water wires

We isolated water-wire trajectories from all-atom 10ns MD simulations of water permeation through AQP1 using TCL scripts written and executed using Visual Molecular Dynamics (VMD) molecular graphics software. We create an ensemble of water wires from ~30,000 trajectories thereby sampling a large number of possible geometries of the single file of water through AQP1. We observed that all the water wires assume a spiral path which can be approximated to an elliptical helix. To this end, we performed a *k*-means clustering[1] over the ensemble of such helices on the three parameters, (1) major axis, (2) minor axis of the ellipse, and (3) pitch per turn of the helix, to group similar water wire geometries. Each water wire was represented as a point in a 3-dimensional space with coordinates equal to the magnitude of the three parameters respectively. *K* clusters were generated by assigning each data point to a cluster whose center is nearest to the data point. *K* is incremented over successive iterations and the highest intra-cluster variance is stored for every *k*. In factor analysis, scree plot[2] is a graphical representation of intra-cluster variance against a series of sequential cluster levels, where an appropriate number of clusters is defined as one at which the reduction of the variance slows significantly. Next, we used a scree plot to identify the optimal number of clusters (*k*) for which the maximum intra-cluster variance in minimum. We identified four unique water wire geometries through AQP1 ($k^{optimal}$=4) represented by four clusters (Supplementary Figure 1 and Supplementary Table 1). Thereafter, we selected the water wire closest to each cluster center as the "representative" wire for that geometry. Using the IPRO[3] input language terminology in PoreDesigner, the protein molecule permitted to mutate is referred to as design molecule (DM) and the molecule they bind is referred to as target molecule (TM). Within the DM, the exact residues that are allowed to mutate are called design positions (DPs). In PoreDesigner, we declared the four water wires as target molecules (TMs) while the OmpF (2omf.pdb) and 25 of its pore constricting residues were defined as design molecules (DM) and design positions (DPs) respectively. We ran PoreDesigner for all four water wires (from *k*-means clustering) to identify convergent designs which are independent of the permeating water wire geometry.

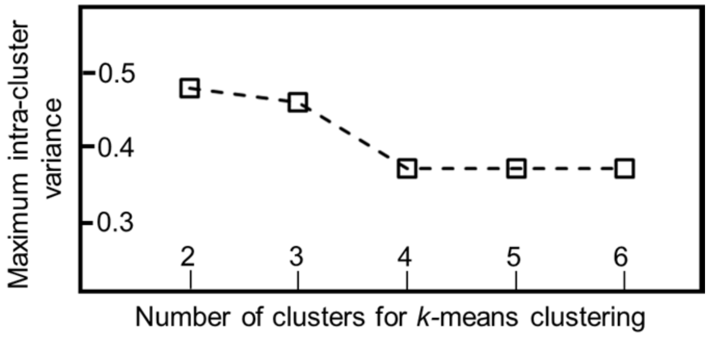

Supplementary Figure 1. Scree plot showing the maximum intra-cluster variance against the value *k*. Here *k* is the number of clusters. Each cluster contains geometrically similar water wires. Each water wire is represented by a point in the 3D space with coordinates (R_1, R_2, and C). Two such proximal points are assigned membership to the same cluster. The plot reveals that there are four unique geometries that water assumes while permeating through AQP1.

Supplementary Table 1. The parametric equation of the four characteristic water wires from four clusters. One water wire from each of the four clusters reveal the possible geometries water may take during pressure driven flow through the AQP1 channel.

Water wire cluster no.	Parametric equation of representative water wires from each cluster
1	$x = 0.401\ sin\theta, y = 0.121\ cos\theta, z = 2.31\ \theta$
2	$x = 0.321\ sin\theta, y = 0.281\ cos\theta, z = 3.24\ \theta$
3	$x = 0.314\ sin\theta, y = 0.309\ cos\theta, z = 1.81\ \theta$
4	$x = 0.319\ sin\theta, y = 0.277\ cos\theta, z = 2.35\ \theta$

Stepwise description of PoreDesigner

PoreDesigner uses results from molecular dynamics simulations (all atom 10ns with 2 fs timestep) of pressure driven water transport through tetrameric AQP1-membrane assembly. The tetrameric AQP1 contributes four water wires per frame. Thus, 30,000 frames were used to glean ~1,20,000 water wires. However, nearly 20,000 wires could not be used for the analysis because they were incomplete (i.e. a gap of more than 4Å between any two contiguous water molecules).

Preparatory phase to *k*-means cluster 1,000,000 water wires

In order to discern the principal geometric modes of water permeation through AQP1, each water wire was fit to an elliptic helix equation. The parametric form of an elliptical helix is:$x = R_1 \cos\theta; y = R_2 \sin\theta; z = C\theta.$

A *k*-means clustering was performed on the semi major (R_1), semi minor (R_2) axes, and the pitch per turn (C) for one million generated elliptical helices to identify the different geometries of the water wire.

1.2 Aligning OmpF structure and placing water wires

The following sub-steps were performed to prepare the OmpF molecule for redesign.

Step 1: The OmpF (2omf.pdb) structure was oriented such that the pore axis coincides with the Z-axis.

Step 2: The OmpF molecule was translated along the Z-axis to place the origin at the pore constriction center.

Step 3: The four water wires were placed one at a time in the channel cavity of OmpF such that the vertical axis of symmetry of the water wire helix coincides with the Z-axis (pore axis).

Step 4: The OmpF molecule was used as the design molecule (DM) and the water wire as the target molecule (TM) and the 25 pore constricting residues were defined as design positions (DPs) in the IPRO[3] input file used in PoreDesigner. In IPRO DMs are the protein(s) that (may) undergo mutations, TMs are the molecule(s) that bind to the DMs and DPs are the list of residue positions in DPs that are allowed to mutate.

OmpF redesign phase

In this phase, the OmpF DPs are allowed to mutate to create a narrow yet hydrophobic pore such that the resultant pore allows a single-file water transport at enhanced hydraulic permeation rates. A set of nine amino acids were chosen (Trp, Phe, Tyr, Ile, Met, Leu, Pro, Val, and Ala) as allowed redesign choices.A minimum of 50% of the newly introduced residues had to be either Trp, Phe or Tyr. This sped up the identification of acceptable designs as PoreDesigner would not have to "sift" through all possible

combinations of smaller allowed amino acids (with low interaction energy with the water wire) which would ultimately be rejected as they did not meet the pore size cutoffs.

PoreDesigner iterative redesign cycle

The PoreDesigner iterative redesign cycle largely follows the sequence of steps defined in IPRO[3].

<u>*Step 1:*</u> Backbone perturbation of an 11-amino acid window with a randomly chosen DP as the sixth (central) amino acid is performed. The side chains are stripped, and backbone phi and psi dihedral angles are randomly perturbed using values from a normal distribution (μ=0, σ=1.5º).

<u>*Step 2:*</u> Repacking the amino acids side chains inside and within 4.5Å of the perturbed region and redesign of all DPs included within the perturbed region to any of the allowed amino acids is done. This optimization step is carried out by solving an MILP problem with an objective function of maximizing the interaction energy (van der Waals, electrostatics, and solvation).Constraints in the MILP formulation impose selection of only one amino acid rotamer at each design position, mutationof at least 50% of the DPs to longer side chain amino acids (Trp, Phe, Tyr, and Met), and prevention of the same amino acid-rotamer combination to be chosen at the same design position in follow up iterations.

This MILP formulation that is solved in Step 2 is stated as follows:

Sets:

i,j =1, …, N, set of all design positions

r,s=1, …, R, set of rotamers for position i.

$U=\{(i,r)|i = 1,\dots,N; r = 1,\dots,R\}$ universal set of all feasible residue position and amino acid rotamer combinations.

$CUTS = \{(i,r)|y_{ir} = 1\}$ set of residue position and amino acid rotamer combinations for the design obtained from any iteration

A={Trp, Tyr, Phe, Met,Ile, Leu, Val, Pro, Ala} set of allowed amino acids

U_{AA}= set of all amino acids

Parameters:

EC_{ir} stores the interaction energy of rotamer r at position i and the non-rotamer region

ER_{ir}^{js} stores interaction energy betweenrotamer r at position i and rotamer s at position j

$$LONG_r = \begin{cases} 1, \text{if } r \text{ is a rotamer of a long side chain amino acid (Trp, Phe, Tyr, Met)} \\ 0, \quad \text{otherwise} \end{cases}$$

$$SHORT_r = \begin{cases} 1, \text{if } r \text{ is a rotamer of a short side chain amino acid (Ile, Leu, Val, Pro, Ala)} \\ 0, \quad \text{otherwise} \end{cases}$$

$M =$ maximum number of mutations allowed at each iteration.

$$WT_{ir} = \begin{cases} 1, \text{if rotamer } r \text{ is seen at design position } i \text{ in the wild type structure} \\ 0, \quad \text{otherwise} \end{cases}$$

Binary variables:

$$y_{ir} = \begin{cases} 1, & \text{if rotamer } r \text{ is selected at position } i \\ 0, & \text{otherwise} \end{cases}$$

$$w_{ir}^{js} = \begin{cases} 1, & \text{if rotamer } r \text{ is selected at position } i \text{ and } s \text{ at position } j \\ 0, & \text{otherwise} \end{cases}$$

$$z_{ir} = \begin{cases} 1, & \text{if rotamer } r \text{ is selected with amino acid from set } A \text{ at position } i \text{ upon mutation} \\ 0, & \text{if position } i \text{ is unmutated} \end{cases}$$

MILP formulation

$$Maximize \sum_{i=1}^{N} \sum_{r=1}^{R_i} y_{ir} EC_{ir} + \sum_{i=1}^{N-1} \sum_{r=1}^{R_i} \sum_{j=i+1}^{N} \sum_{s=1}^{R_j} w_{ir}^{js} ER_{ir}^{js}$$

subject to:

$$\sum_{r=1}^{R} y_{ir} = 1, \ \forall i |\ 1, \ldots, N \tag{1}$$

$$y_{ir} = \sum_{s=1}^{R} w_{ir}^{js}, \ \forall i | 1, \ldots, N-1, \forall j | i+1, \ldots, N, \forall r | 1, \ldots, R \tag{2}$$

$$y_{js} = \sum_{r=1}^{R} w_{ir}^{js}, \ \forall i | 1, \ldots, N-1, \forall j | i+1, \ldots, N, \forall s | 1, \ldots, R \tag{3}$$

$$\sum_{i=1}^{N} y_{ir} LONG_r - \sum_{i=1}^{N} y_{ir}\, SHORT_r \geq 0, \qquad \forall r \in A \tag{4}$$

$$\sum_{r=1}^{R} \sum_{i=1}^{N} z_{ir} \leq M \tag{5}$$

$$\sum_{r=1}^{R} z_{ir} \leq 1, \forall i | 1, \ldots, N \tag{6}$$

$$z_{ir} = 0\,, \forall i | 1, \ldots, N, \forall r \in U_{AA} \backslash A \tag{7}$$

$$y_{ir} \geq z_{ir}\,, \forall i | 1, \ldots, N, \forall r | 1, \ldots, R \tag{8}$$

$$y_{ir} \geq \left(1 - \sum_{r=1}^{R} z_{ir}\right) WT_{ir} \tag{9}$$

$$\sum_{\substack{r=1 \\ (i,r) \in CUTS}}^{R} \sum_{i=1}^{N} y_{ir} + \sum_{\substack{r=1 \\ (i,r) \in U \backslash CUTS}}^{R} \sum_{i=1}^{N} (1 - y_{ir}) \leq (R \times N) - 1 \tag{10}$$

The objective function maximizes the net interaction energy of the rotamers with the non-rotamer portion of the binding assembly and with each other. Constraint 1 ensures that exactly one rotamer is selected at each design position. Constraints 2 and 3 ensure that w_{ir}^{js} is one only when both y_{ir} and y_{js} have a value of one. Constraint 4 ensures that at least 50% of the DPs are mutated to longer side chain residues (Trp, Phe, Tyr, and Met). This alleviates the need to cycle through designs with all smaller side chain residues (Ile, Leu, Val, Pro, and Ala) and this accelerates convergence. Using a very low percentage will result in longer run times owing to small side chain-rich designs being identified first which will be weaned out at the pore size check at *step 5*. On the other hand, a very high percentage will result in steric clashes between the chosen long side chain residues yielding larger than expected pore sizes (akin to OCDTFTrp design). Constraint 5 ascertains that at most M out of N design positions are allowed to mutate in a given iteration. The value of M is randomly generated for each PoreDesigner iteration and fed as a parameter to the MILP step. Constraint 6 makes sure that if a design position is to be mutated, it assumes only one new rotamer and if unmutated it retain the wild type amino acid rotamer while constraint 7 prevents mutation to non-hydrophobic residues. Constraints 8 and 9 together pass on the information about the current design to the objective function using the binary variable y_{ir}. If a design position is mutated, constraint 8 uses z_{ir} to set the y_{ir} value for that position and rotamer combination to one. However, if a design position is not mutated (i.e. $z_{ir} = 0$), constraint 9 uses parameter WT_{ir} to set the y_{ir} value to one corresponding to the wild-type configuration of that residue. Therefore, an MILP design has y_{ir} values of one for each design position obtained either from a mutation or from the wild type configuration (if unmutated). At the end of each iteration the design is appended to a CUTS set. Constraint 10 makes sure none of the existing designs from the CUTS set are chosen in the current iteration.

Step 3: A local, rigid-body docking of the water wire using random translations in the X, Y, and Z directions by sampling coordinates for the water wire from a normal distribution centered at zero and standard deviations of 0.2Å, 0.2Å, and 2Å, respectively.

Step 4: A force field complex energy minimization in Cartesian coordinates x,y,z using a gradient based search.

Step 5: Pore size analysis is performed using a PoreAnalyzer module (details in section 3) to check if it satisfies the desired pore size. The interaction energy is calculated if the pore opening is within the desired size range, otherwise the design is discarded.

Step 6: If the design is accepted in the previous step, the interaction energy between the water wire and the redesigned OmpF is calculated. Redesigns with lower interaction energy than the currently best are always accepted. Redesigns with larger interaction energies (in absolute magnitude) are accepted with a probability or a Boltzmann factor equal to $e^{\frac{-\Delta(interaction\ energy)}{kT}}$ (i.e., Metropolis criterion where, k is the Boltzmann constant and is ~0.33×10^{-23}cal/K, and T is the temperature in K).A temperatureof 3,640 K in the Boltzmann factor is used which ensures that there is a 25% probability that a redesign with an interaction energy 10 kcal/mol more negative than the best so far will be retained.

Step 7: A cumulative set of integer cuts which stores information about the current mutation (residue, position, rotamer). This ensures that no redesign (either accepted or rejected previously) is revisited.

Step 8: A number of perturbation/redesign iterations of PoreDesigner are performed until a pre-specified number of accepted redesigns in terms of pore size are retrieved (i.e., typically we require 30 accepted redesigns).

Post-redesign analysis of results

This step is used to estimate the pore constriction diameter of the designed OmpF mutant and accept or discard designs accordingly.

Step 1: Introduce in the ~8 Å long constriction region (Supplementary Figure 2) perpendicular planes at every 0.5 Å (approximately 16 slices).

Step 2: Apply developed PoreAnalyzer algorithm comprised of the following sub-steps:

1. Supply the oriented OmpF pdb structure.
*2.*Identify the list of pore center coordinates at each of the 16 slices (Supplementary Figure 2) of the pore constriction region.
3. At each slice, find the coordinates of the pore constricting atoms (including their van der Waals radii) nearest to the pore center coordinates.
4. At each slice, fit the largest ellipse (Ellipse fitting method for details) that just touches the pore constricting atoms (with no atoms inside the ellipse). The ellipse is centered at the pore center coordinates for a given slice.
5. Store the major axis dimensions (D_i) of the ellipses from each slice in a list D^{pore} such that, $D_i \in D^{pore}$. The complete set of major axes for the ~8Å constriction region is stored in D^{pore}.
*6.*The minimum value in D^{pore}(i.e. $_i Min\ D^{pore} = D^{pore}_{min}$ determines the actual pore bottleneck diameter or the pore constriction diameter (Supplementary Figure 2).
7. The minor axis dimension corresponding to the ellipse for which major axis is equal toD^{pore}_{min} is identified and thus the pore constriction dimensions are determined.

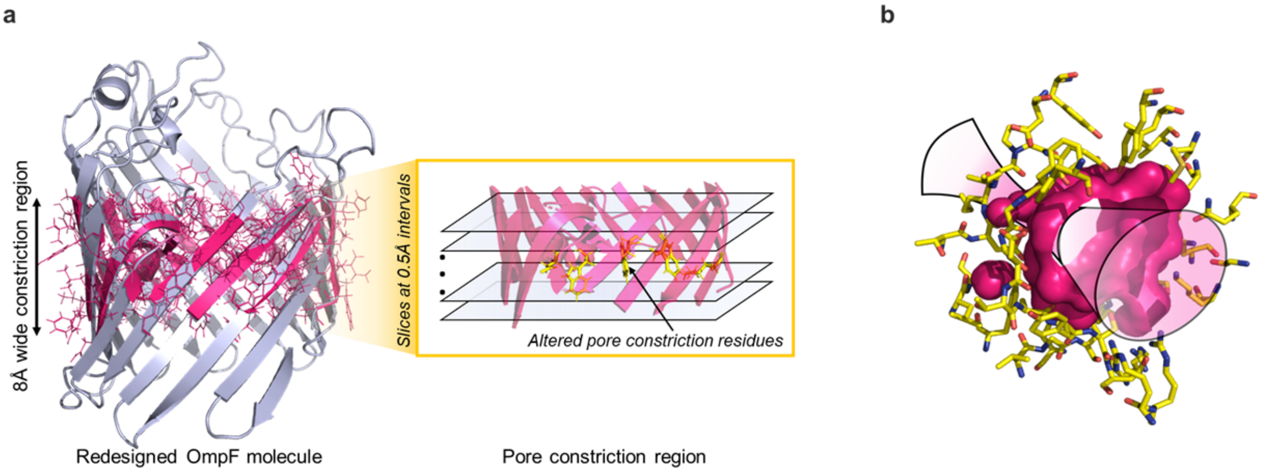

Supplementary Figure 2 (a) The 8 Å constriction region is divided into slices every 0.5 Å. Pore area is calculated at each slice and the lowest of them determines the redesigned pore constriction area. **(b)**The OmpF pore profile was generated using PoreAnalyzer module and visualized using PyMOL. The twenty-five pore constriction residues are highlighted in yellow. The channel cavity (pink) is composed of multiple pink spheres placed at regular intervals of 0.5 Å. A schematic hour-glass shaped internal pore geometry has been overlaid.

Step 3: Impose a design check to retain OmpF redesigns if it meets the desired pore size criteria. The first check is imposed while designing AQP-like small pores (<4 Å diameter) that allow single file water transport. The latter is used for redesigning OmpF for selective separation (rejects aqueous solute A but not B) of aqueous solutes (A and B) with hydrodynamic diameters D_A and D_B respectively.

Check 1: Accept the design if$D^{pore}_{min} < 4$ Å is satisfied.

Check 2: Accept the design if both $D^{pore}_{min} < D_A$and $D^{pore}_{min} > D_B$ are satisfied.

Accepted OmpF designs are sorted in decreasing order of the DM-TM interaction energy implying that redesigns with least interaction with the water wire are ranked higher. Thus, PoreDesigner

can be used to create the selective internal structure of AQP1 (or any desired pore size) inside the stable beta-scaffold of OmpF.

Ultimately, we obtain the structures for the final 100 frames of MD simulation of pressure driven water transport through OmpF and the mutant designs. We use the PoreAnalyzer module on them to report the predicted pore sizes. This is to ensure we track any pore widening that might occur during water permeation.

Ellipse fitting method

At each slice of pore contriction region we fit the general equation of a rotated ellipse $Ax^2 + Bxy + Cy^2 + Dx + Ey + F = 0$ using non-linear regression. Here, the coefficients A, B, C, D, E, and F represent arbitrary real-valued constants with at least one of A, B, or C as well as at least one of D, E, or F nonzero. Although all conic sections can be represented in this way, some combination of the constants could give rise to one of the five degenrate conic sections (a point, a line, or two intersecting lines, two parallel lines or the empty set). But we verified that resulting figure is a non-degenerate conic section (circle or ellipse) by checking that the area inside the curve after the non-linear regression was less than 60. This is because the wild type OmpF pore area with major and minor axes lengths 11 Å and 7 Å respectively, is 60.47 Å^2.

Inner, outer pore wall and overall hydrophobicity scores

We used the $\Delta \boldsymbol{G}_{\boldsymbol{transfer}}^{\boldsymbol{water \to ethanol}}$ values[4] (from the Kyte-Doolittle (KD) hydrophobicity scale) to evaluate the inner pore wall, outer pore wall and overall KD-hydrophobicities of the OCD-TFTrp, UCD, and CSD designs which were subsequently purified and expressed, and embedded in vesicles for transport experiments. KD-hydrophobicities have been used as a standard to assess the performance of novel hydrophobicity scales by Perunov *et al.*[5]. Furthermore, Kister *et al.*[6] has reported that the accuracy of the KD scale in estimating protein hydrophobicities is considerably reliable. Each of the three hydrophobicity scores were calculated by adding the products of amino acid (i) frequencies (n_i) to their individual $\Delta \boldsymbol{G}_{\boldsymbol{transfer}}^{\boldsymbol{water \to ethanol}}$ values. Amino acid frequencies refer to the number of times (n_i) a single amino acid$i$ occurs. The overall hydrophobicity score of a channel protein is the sum of its inner pore wall and outer pore wall hydrophobicity scores calculated from transfer free energies (equation 11).

$$\sum_{i=1}^{20} \left(n_i^{inner\_porewall} \times \Delta \boldsymbol{G}_{\boldsymbol{transfer}}^{\boldsymbol{water \to ethanol}}{}_i \right) + \sum_{i=1}^{20} \left(n_i^{outer\_porewall} \times \Delta \boldsymbol{G}_{\boldsymbol{transfer}}^{\boldsymbol{water \to ethanol}}{}_i \right) = Overall\ hydrophobicity\ score \tag{11}$$

In order to decide if a given amino acid is a part of the inner pore wall or the outer pore wall, Python 2.7 scripts were written. If the distance between the pore center and the Cα atom of any amino acid is greater than that from its Cβ atom (or analogous atom), then it is counted as an inner pore wall residue. Otherwise, it is counted an outer pore wall residue.

Molecular Dynamics methods

All MD simulations were performed using the program NAMD[7], a 2 fs integration time step, and 2–2–6 multiple time-stepping. Parameters for the POPC lipid-bilayer, OmpF protein, and ions were taken from the CHARMM36 parameter set with the CMAP corrections[8]. A TIP3P model was used for water[9]. All simulations employed a 10–12 Å cutoff for van der Waals and short-range electrostatic forces, the particle mesh Ewald (PME) method for long-range electrostatics[10] computed over a 1.1 Å grid and periodic

boundary conditions. Simulations in the NPT (constant number of particles N, pressure P, temperature T) ensemble were performed using a Lowe–Andersen thermostat[11] and Nosé–Hoover Langevin piston pressure control[12] set at 295 K and 1 atm, respectively. Visualization and analysis were performed using VMD[13].

The wild type OmpF protein was obtained from protein database with the accession code, 2OMF[14]. Mutant OmpF proteins were obtained from the PoreDesigner algorithm. The POPC lipid bilayer was built using the Membrane Builder plugin in VMD[13]. An OmpF timer was subsequently inserted into the lipid membrane and any overlapping lipids were deleted or removed to maintain the area per lipid at a constant value (70 Å^2). Two sets of systems were created for the wild type and each OmpF mutants; the first set had a solution of Na^+ ions to generate an electrically neutral system and a second set contained a solution of 1M NaCl. Thereafter a total of eight systems were constructed, each measuring 14.3 nm×14.3 nm×7.2 nm. A periodic boundary was employed along the xy-plane and the system comprised 111,500 atoms.

After assembly, each system was energy minimized for 5000 steps and equilibrated for 45 ns in the NPT ensemble. To induce an ionic current, systems containing 1M NaCl were simulated for an additional 35 ns in the presence of a uniform electric field. A 500 mV voltage bias produced by the field follows $V = -L_z E_z$, where L_z is the length of the unit cell along the z-axis[15,16]. All simulations in the presence of an electric field (~0.1538 kcal/ (mol Å *e*)) were simulated in an NVT (constant number of particles N, volume V, temperature T) ensemble.

Calculation of the osmotic permeability followed a method established in a previous work[17] utilizing the collective diffusion model developed by Zhu, *et al*[18]. Permeability analysis was performed in the last 30 ns of the 35 ns equilibrium trajectories of systems in solutions containing only Na^+ ions. To this end, diffusion coefficient, D, of water molecules moving through each channel was computed before estimating the osmotic permeability as $P_f = v_W \times D$ where, v_W is the volume of a single water molecule.

Ultimately, the conductance through the channels were calculated using standardized methods measuring the displacement of ions through the protein channel during the last 25 ns of the 35 ns simulations of the systems subject to an applied external electric field[16].

Estimation of maximum OmpF conductances during salt removal

In this calculation example, we used wild type OmpF as an example to discuss based on the single channel (protein) permeability and simulated protein conductance, what conductance value would lead to drinking water quality for a seawater feed and then we extend this analogy to find similar results for other feed types. The overall approach is to assume the thermodynamic equivalency of the electrochemical driving force for which conductance measurement was obtained to the concentration driven driving force that would drive ions across the membrane. In summary, from this approximate analysis, we find that a conductance as low as ≤0.018 nS would be needed for seawater desalination (assuming a feed of 35 g/L), ≤ 0.034 nS for brackish water desalination (assuming a feed of 5 g/L), and ≤ 0.056 nS for treating low salinity wastewater (assuming a feed salinity of 2g/L). All these targets can be possibly met by the UCD and OCD designs as their simulated conductances lie within the range simulated for these mutants. An example calculation is described in detail below.

For the seawater case, the salinity (NaCl mass concentration) on the feed side is 35g/L while the salinity (NaCl mass concentration) on the permeate side is 0.5g/L (which is the salinity of drinkable water) as shown in the the following figure. Due to the concentration difference between feed side and the permeate side, there will be NaCl flux across the membrane, defined as the flux J_{NaCl}. We also assume that the molar ratio of Na+ and Cl- ions in the feed is approximately 1:1.

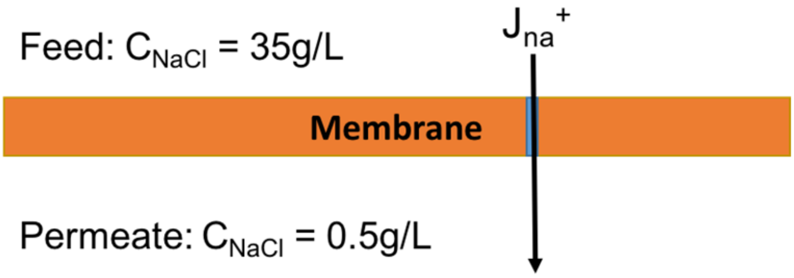

Now, we consider a situation that applying an electric field on the opposite direction (which means there is high voltage on the permeate side while low voltage on the feed side) to create driving force thermodynamically equivalent to the expected solute (Na^+) passage under a concentration gradient condition. A similar hypothetical analysis could also be conducted for Cl^- in the opposite direction.

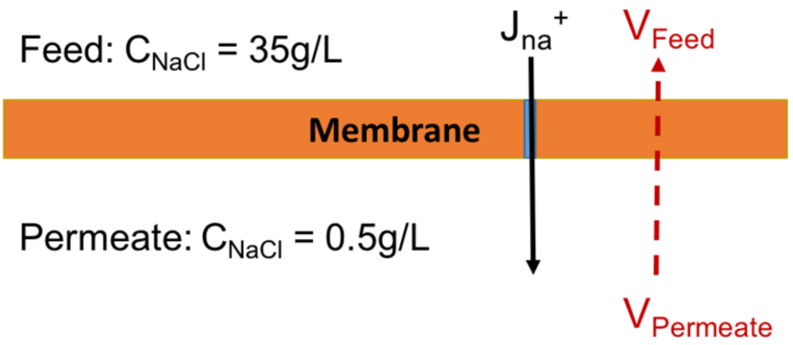

Based on the Nernst-Planck equation:

$$J_{net} = J_{Na} + J_{ele} = -D\frac{dC}{dx} - uCzF\frac{dV}{dx} \tag{12}$$

where D is the diffusion coefficient of the solute, u is the mobility of an ion, and D = uRT based on Einstein relation.

Thus,

$$J_{net} = J_{Na} + J_{ele} = -uRT\frac{dC}{dx} - uCzF\frac{dV}{dx} \tag{13}$$

when the net flux J_{net} is zero, $J_{Na} = -J_{ele}$:

$$\frac{dV}{dx} = -\frac{RT}{zF}\frac{dlnC}{dx} \tag{14}$$

$$\Delta V^* = V_{feed} - V_{permeate} = -\frac{RT}{zF}ln\frac{C_{feed}}{C_{permeate}} \tag{15}$$

Based on the Na^+ conductance results from simulation, we calculated the ion (Na^+) transport rate:

$$I^* = V * G = \left(-\frac{RT}{zF}ln\frac{C_{feed}}{C_{permeate}}\right) * G \tag{16}$$

Under this condition, it means when the voltage difference between feed side to permeate side is ΔV^* on the membrane model above, there will be no Na^+ flux across the membrane, since the feed side and permeate side reach chemical potential equilibrium. And based on the conductance simulation results, the ion (Na^+) transport rate that provides the 54mV voltage difference is I^*. Thus, when there is no electric field applied across the membrane, J_{net} is maximum, and the Na^+ flux equals to the ion transport rate due to a ΔV^* voltage difference. So the maximum ion transport rate is I^* when the salinity on the feed side is 35g/L while the salinity on the permeate side is 0.5g/L (assuming an equimolar mixture of Na^+ and Cl^-). Based on the single OmpF (wild type) permeability p_f that's measured in this paper, so when there is no voltage applied, the molar ratio of water transport rate to NaCl transport rate is $\frac{\mathrm{pf}}{I^*}$, which equals to 1.94 % (w/w) Na^+ in the product solution. Thus, on the permeate side, we will end up with 1.94 g Na^+/L water when operating a OmpF (wild type) based membrane, compared to 21.2 g/L Na^+ on the feed side.

Using the above analysis, we can calculate at what maximum Na^+ conductance value (Supplementary Table 2), we may expect to see sufficient salt rejection for seawater desalination leading to a sodium concentration equivalent to a 0.5 g/L NaCl permeate by simply scaling the WT conductance values that provides the above concentration to the desired Na+ value in permeate (~ 0.303 g/L of Na+). We can do a similar analysis to find the maximum Cl^- conductance value that would lead to the 0.5 g/L NaCl product for chloride. Upon adding the two values we find that a value of .018 nS/cm would be sufficient if the WT water permeability (the lowest OmpF measured among the mutants) is assumed. This conductance value is within the range of values estimated using simulations for the UCD and OCD mutants and thus we could assume that both the UCD and OCD mutants would be expected to be a key transport element of a successful OmpF mutant based biomimetic desalination membrane if synthetic hurdles are overcome. Net permeability of OmpF and its mutants in reconstituted biomimetic membranes have been illustrated in Supplementary Figure 4.

Supplementary Table 2. Maximum calculated conductance values for achieving drinking water quality values in permeate from hypothetical OmpF mutant membranes

		Na^+ conductance (0.6nS)		Cl^- conductance (0.26nS)	
Water type	Salinity (g/L)	Final Na+ conc of permeate (g/L	Ideal Na^+ conductance	Final Cl- conc of permeate (g/L)	Ideal Cl^- conductance
Seawater	35	19.4	0.009	5.5	0.009
Brackish water	5	10.6	0.017	3	0.017
Waste water	2	6.4	0.028	1.8	0.028

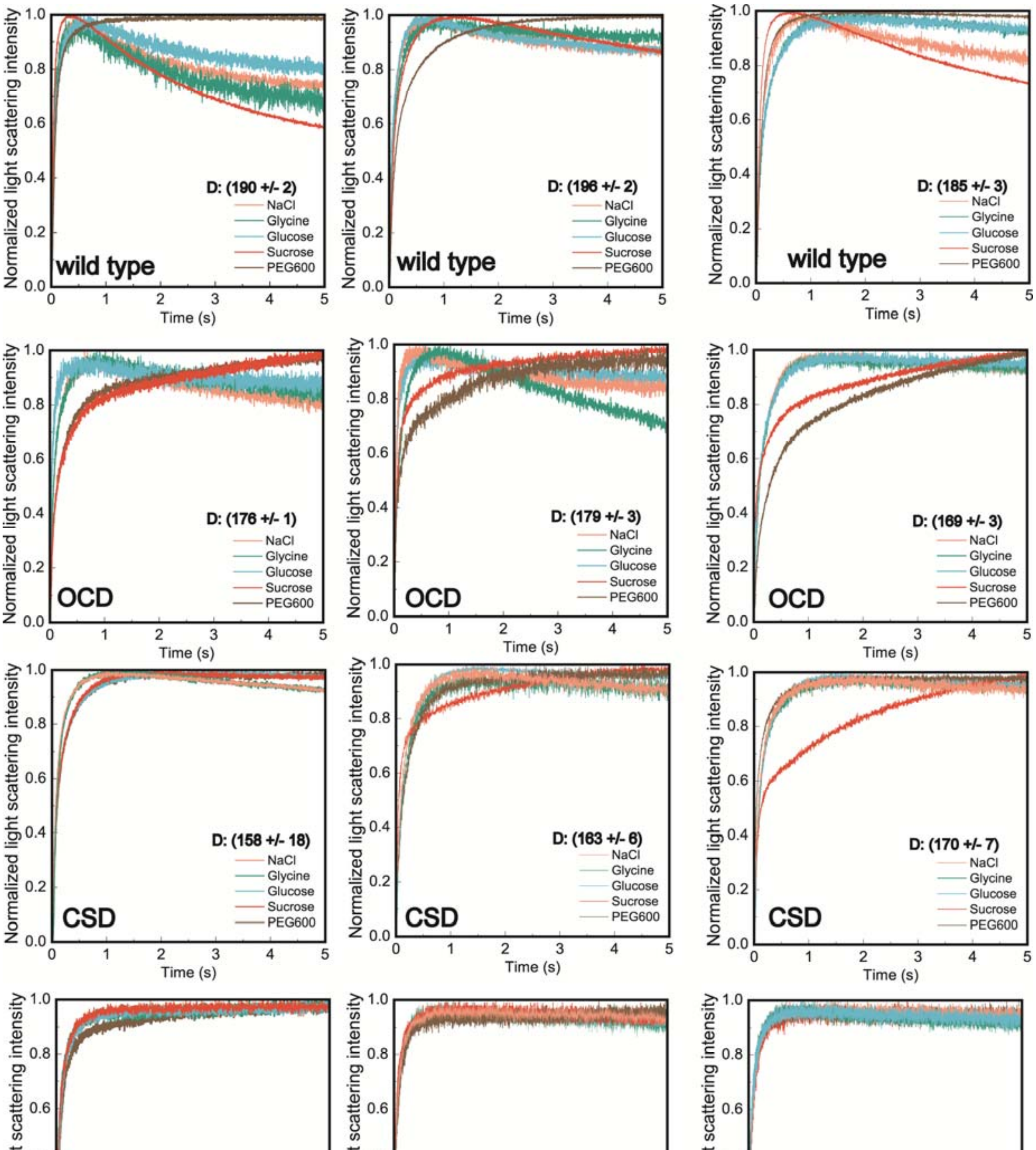

Supplementary Figure 3. Complete stopped flow results for OmpF wild type and its mutants solute rejection determination: wild type OmpF (WT) showed rejection of PEG600, OCD design OmpF mutant showed rejection of sucrose, CSD design OmpF mutant showed rejection of glucose and UCD design OmpF mutant showed rejection of NaCl. The average vesicle diameter (D in nm) have been shown in each panel. Overall, the average size diameter in the stopped flow experiments is 175 (±13) nm.

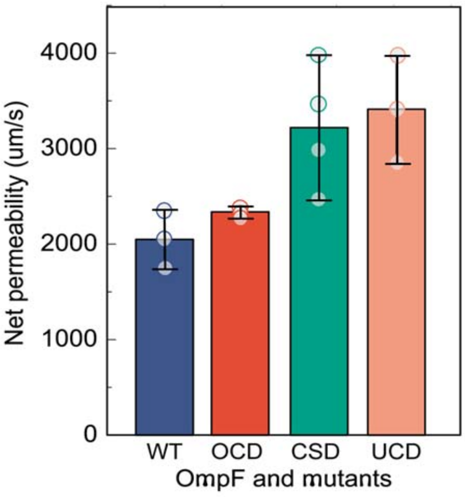

Supplementary Figure 4: Net permeability of OmpF wild type and its mutants reconstituted in biomimetic membranes.

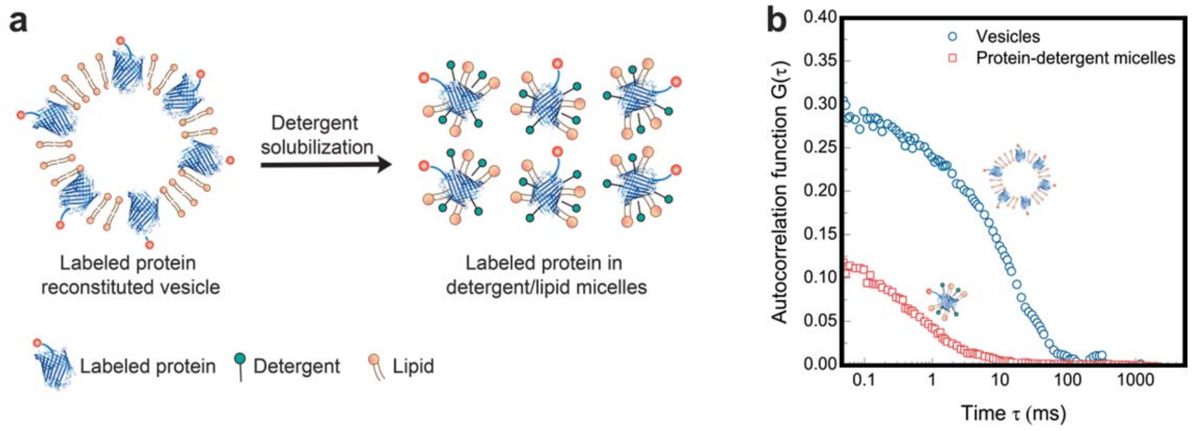

Supplementary Figure 5: Application of Fluorescence Correlation Spectroscopy (FCS) to determine the number of proteins per vesicle (N_{pro}/N_{ves}). **(a)**: Add detergent to break down labeled protein reconstituted vesicles into micelles. **(b)** Autocorrelation function of vesicles and micelles (from FCS measurements)

>2omf

AEIYNKDGNKVDLYGKAVGLHYFSKGNGENSYGGNGDMTYARLGFKGETQINSDLTGYGQWEYNFQGNNSEGADAQTGNKTRLAF
AGLKYADVGSFDYGRNYGVVYDALGYTDMLPEFGGDTAYSDDFFVGRVGGVATYRNSNFFGLVDGLNFAVQYLGKNERDTARRSN
GDGVGGSISYEYEGFGIVGAYGAADRTNLQEAQPLGNGKKAEQWATGLKYDANNIYLAANYGETRNATPITNKFTNTSGFANKTQDV
LLVAQYQFDFGLRPSIAYTKSKAKDVEGIGDVDLVNYFEVGATYYFNKNMSTYVDYIINQIDSDNKLGVGSDDTVAVGIVYQF

>CSD

AEIYNKDGNKVDLYGKAWGWHYFSKGNGENSYGGNGDWTWARLGFKGETQINSDLTGYGQWEYFFQGNNSEGADAQTGNKTWL
AFAGLKYADVGSFDYGRNYGVVYDALWYTDMLPAFWGDTWYWDDFFVFRVGGVATYRNSNFFGLVDGLNFAVQYLGKNERDTAR
RSNGDGVGGSISYEYEGFGIVGAYGAADRTNLQEAQPLGNGKKAEQWATGLKYDANNIYLAANYGETRNATPITNKFTNTSGFANKT
QDVLLVAQYQFDFGLRPSIAYTKSKAKDVEGIGDVDLVNYFEVGATYYFNKNMSTWVDYIINQIDSDNKLGVGSDDTVAVGIVYQF

>UCD

AEIYNKDGNKVDLYGKAWGWHYFSKGNGENSYGGNGDWTWARLGFKGETQINSDLTGYGQWPYWFQGNNSEGADAQTGNKTW
LAFAGLKYADVGSFDYGRNYGVVYDALWYTWMLPWGWGDTWYSDDFFVFRVGGVATYRNSNFFGLVDGLNFAVQYLGKNERDTA
RRSNGDGVGGSISYEYEGFGIVGAYGAADRTNLQEAQPLGNGKKAEQWATGLKYDANNIYLAANYGETRNATPITNKFTNTSGFANK
TQDVLLVAQYQFDFGLRPSIAYTKSKAKDVEGIGDVDLVNYFEVGATYYFNKNMSTGVDYIINQIDSDNKLGVGSDDTVAVGIVYQF

>OCDTFTrp

AEIYNKDGNKVDLYGKAWGWHYFSKGNGENSYGGNGDWTWAWLGFKGETQINSDLTGYGQWWYWFQGNNSEGADAQTGNKT
WWAFAGLKYADVGSFDYGRNYGVVYDAWWYTWWWPWWWWWWTWWWDDFFWWWVGGVATYRNSNFFGLVDGLNFAVQYLGK
NERDTARRSNGDGVGGSISYEYEGFGIVGAYGAADRTNLQEAQPLGNGKKAEQWATGLKYDANNIYLAANYGETRNATPITNKFTNT
SGFANKTQDVLLVAQYQFDFGLRPSIAYTKSKAKDVEGIGDVDLVNYFEVGATYYFNKNMSTYVDYIINQIDSDNKLGVGSDDTVAVGI
VYQF

```
               1                           30                           60

2omf           AEIYNKDGNKVDLYGKAVGLHYFSKGNGENSYGGNGDMTYARLGFKGETQINSDLTGYGQ
CSD            AEIYNKDGNKVDLYGKAWGWHYFSKGNGENSYGGNGDWTWARLGFKGETQINSDLTGYGQ
UCD            AEIYNKDGNKVDLYGKAWGWHYFSKGNGENSYGGNGDWTWARLGFKGETQINSDLTGYGQ
OCDTFTrp       AEIYNKDGNKVDLYGKAWGWHYFSKGNGENSYGGNGDWTWAWLGFKGETQINSDLTGYGQ

2omf           WEYNFQGNNSEGADAQTGNKTRLAFAGLKYADVGSFDYGRNYGVVYDALGYTDMLPEFGG
CSD            WEYFFQGNNSEGADAQTGNKTWLAFAGLKYADVGSFDYGRNYGVVYDALWYTDMLPAFWG
UCD            WPYWFQGNNSEGADAQTGNKTWLAFAGLKYADVGSFDYGRNYGVVYDALWYTWMLPWGWG
OCDTFTrp       WWYWFQGNNSEGADAQTGNKTWWAFAGLKYADVGSFDYGRNYGVVYDAWWYTWWWPWWWW

2omf           DTAYSDDFFVGRVGGVATYRNSNFFGLVDGLNFAVQYLGKNERDTARRSNGDGVGGSISY
CSD            DTWYWDDFFVFRVGGVATYRNSNFFGLVDGLNFAVQYLGKNERDTARRSNGDGVGGSISY
UCD            DTWYSDDFFVFRVGGVATYRNSNFFGLVDGLNFAVQYLGKNERDTARRSNGDGVGGSISY
OCDTFTrp       WTWWWDDFFWWWVGGVATYRNSNFFGLVDGLNFAVQYLGKNERDTARRSNGDGVGGSISY

2omf           EYEGFGIVGAYGAADRTNLQEAQPLGNGKKAEQWATGLKYDANNIYLAANYGETRNATPI
CSD            EYEGFGIVGAYGAADRTNLQEAQPLGNGKKAEQWATGLKYDANNIYLAANYGETRNATPI
UCD            EYEGFGIVGAYGAADRTNLQEAQPLGNGKKAEQWATGLKYDANNIYLAANYGETRNATPI
OCDTFTrp       EYEGFGIVGAYGAADRTNLQEAQPLGNGKKAEQWATGLKYDANNIYLAANYGETRNATPI

2omf           TNKFTNTSGFANKTQDVLLVAQYQFDFGLRPSIAYTKSKAKDVEGIGDVDLVNYFEVGAT
CSD            TNKFTNTSGFANKTQDVLLVAQYQFDFGLRPSIAYTKSKAKDVEGIGDVDLVNYFEVGAT
UCD            TNKFTNTSGFANKTQDVLLVAQYQFDFGLRPSIAYTKSKAKDVEGIGDVDLVNYFEVGAT
OCDTFTrp       TNKFTNTSGFANKTQDVLLVAQYQFDFGLRPSIAYTKSKAKDVEGIGDVDLVNYFEVGAT

2omf           YYFNKNMSTYVDYIINQIDSDNKLGVGSDDTVAVGIVYQF
CSD            YYFNKNMSTWVDYIINQIDSDNKLGVGSDDTVAVGIVYQF
UCD            YYFNKNMSTGVDYIINQIDSDNKLGVGSDDTVAVGIVYQF
OCDTFTrp       YYFNKNMSTYVDYIINQIDSDNKLGVGSDDTVAVGIVYQF
```

Supplementary Figure 6. The complete fasta sequences and sequence alignments of wild type OmpF and the experimentally tested mutants have been reported. Green segments represent regions conserved in all four proteins and gaps represent a mutation in that amino acid position in at least one mutant.

References:

1. Hamerly, G. & Elkan, C. Learning the k in kmeans. *Adv. neural Inf. Process. …* **17,** 1–8 (2004).
2. Cattell, R. B. The scree test for the number of factors. *Multivariate Behav. Res.* **1,** 245–276 (1966).
3. Pantazes, R. J., Grisewood, M. J., Li, T., Gifford, N. P. & Maranas, C. D. The Iterative Protein Redesign and Optimization (IPRO) suite of programs. *J. Comput. Chem.* **36,** 251–263 (2015).
4. Kyte, J. & Doolittle, R. F. A simple method for displaying the hydropathic character of a protein. *J. Mol. Biol.* **157,** 105–132 (1982).
5. Perunov, N. & England, J. L. Quantitative theory of hydrophobic effect as a driving force of protein structure. *Protein Sci.* **23,** 387–399 (2014).
6. Kister, A. E. & Phillips, J. C. A stringent test for hydrophobicity scales: two proteins with 88% sequence identity but different structure and function. *Proc. Natl. Acad. Sci. U. S. A.* **105,** 9233–9237 (2008).
7. Phillips, J. C. *et al.* Scalable molecular dynamics with NAMD. *Journal of Computational Chemistry* **26,** 1781–1802 (2005).
8. Mackerell, A. D. Empirical force fields for biological macromolecules: Overview and issues. *Journal of Computational Chemistry* **25,** 1584–1604 (2004).
9. Jorgensen, W. L., Chandrasekhar, J., Madura, J. D., Impey, R. W. & Klein, M. L. Comparison of simple potential functions for simulating liquid water. *J. Chem. Phys.* **79,** 926–935 (1983).
10. Darden, T., York, D. & Pedersen, L. Particle mesh Ewald: An N·log(N) method for Ewald sums in large systems. *J. Chem. Phys.* **98,** 10089–10092 (1993).
11. Koopman, E. A. & Lowe, C. P. Advantages of a Lowe-Andersen thermostat in molecular dynamics simulations. *J. Chem. Phys.* **124,** (2006).
12. Martyna, G. J., Tobias, D. J. & Klein, M. L. Constant pressure molecular dynamics algorithms. *J. Chem. Phys.* **101,** 4177–4189 (1994).
13. Humphrey, W., Dalke, A. & Schulten, K. VMD: Visual molecular dynamics. *J. Mol. Graph.* **14,** 33–38 (1996).
14. Berman, H. M. *et al.* The protein data bank. *Nucleic Acids Res.* **28,** 235–242 (2000).
15. Crozier, P. S., Henderson, D., Rowley, R. L. & Busath, D. D. Model channel ion currents in NaCl-extended simple point charge water solution with applied-field molecular dynamics. *Biophys. J.* **81,** 3077–3089 (2001).
16. Aksimentiev, A., Heng, J. B., Timp, G. & Schulten, K. Microscopic kinetics of DNA translocation through synthetic nanopores. *Biophys. J.* **87,** 2086–2097 (2004).
17. Zhu, F., Tajkhorshid, E. & Schulten, K. Collective diffusion model for water permeation through microscopic channels. *Phys. Rev. Lett.* **93,** (2004).
18. Shen, Y. *et al.* Highly permeable artificial water channels that can self-assemble into two-dimensional arrays. *Proc. Natl. Acad. Sci.* **112,** 9810–9815 (2015).
